# Early Response Roles for Prolactin Cortisol and Circulating and Cellular Levels of Heat Shock Proteins 72 and 90**α** in Severe Sepsis and SIRS

**DOI:** 10.1155/2014/803561

**Published:** 2014-08-27

**Authors:** K. Vardas, K. Apostolou, E. Briassouli, D. Goukos, K. Psarra, E. Botoula, S. Tsagarakis, E. Magira, C. Routsi, S. Nanas, G. Briassoulis

**Affiliations:** ^1^First Critical Care Department, Evangelismos Hospital, National and Kapodistrian University of Athens, Ipsilantou 45, 10676 Athens, Greece; ^2^1st Department of Propaedeutic Internal Medicine, Laiko University General Hospital, University of Athens, 17 Agiou Thoma, 115 27 Athens, Greece; ^3^Immunology-Histocompatibility Department, Evangelismos Hospital, Ipsilantou 45, 10676 Athens, Greece; ^4^Department of Endocrinology and Diabetes, Evangelismos Hospital, Ipsilantou 45, 10676 Athens, Greece; ^5^Pediatric Intensive Care Unit, School of Health Sciences, University Hospital, University of Crete, 71110 Heraklion, Greece

## Abstract

*Objective.* To evaluate the early heat shock protein (HSP) and hormonal stress response of intensive care unit (ICU) patients with severe sepsis/septic shock (SS) or systemic inflammatory response syndrome (SIRS) compared to healthy subjects (H). *Methods*. Patients with early (first 48 hrs) SS (*n* = 29) or SIRS (*n* = 29) admitted to a university ICU and 16 H were enrolled in the study. Serum prolactin, cortisol, and plasma ACTH were determined using immunoassay analyzers. ELISA was used to evaluate extracellular HSPs (eHSP90α, eHSP72) and interleukins. Mean fluorescence intensity (MFI) values for intracellular HSPs (iHSP72, iHSP90α) were measured using 4-colour flow-cytometry. *Results*. Prolactin, cortisol, and eHSP90α levels were significantly increased in SS patients compared to SIRS and H (*P* < 0.003). ACTH and eHSP72 were significantly higher in SS and SIRS compared to H (*P* < 0.005). SS monocytes expressed lower iHSP72 MFI levels compared to H (*P* = 0.03). Prolactin was related with SAPS III and APACHE II scores and cortisol with eHSP90α, IL-6, and lactate (*P* < 0.05). In SS and SIRS eHSP90α was related with eHSP72, IL-6, and IL-10. *Conclusion*. Prolactin, apart from cortisol, may have a role in the acute stress response in severe sepsis. In this early-onset inflammatory process, cortisol relates to eHSP90α, monocytes suppress iHSP72, and plasma eHSP72 increases.

## 1. Introduction

Severe sepsis and septic shock are leading causes of death in intensive care units (ICU) worldwide and despite efforts in understanding their pathophysiology and implementing effective treatment, their annual incidence has been projected to increase by 1.5% per year [1]. Sepsis is accompanied by major changes in the hypothalamus-pituitary-adrenal axis through multiple interactions between the autonomous nervous system and immune systems [2]. During early sepsis, initial activation of the pituitary adrenal axis depends on activation of hypothalamus and pituitary by cytokines, while in late sepsis a shift from neuroendocrine to local adrenal regulation of glucocorticoid production has been proposed [3]. The initial release of cytokines from immune cells participates in homeostasis of the body by acting as paracrine, autocrine, and hormonal agents and elevating corticotropin releasing hormone levels (CRH) [3]. Except for CRH, septic patients have increased ACTH, growth hormone, and prolactin levels in the early stage of sepsis [2].

The association of prolactin with modulation of the immune system during sepsis has been studied in septic mice, where administration of prolactin was associated with decreased survival and alterations in immune response [4]. It has been found that prolactin serum levels in critical ill children are usually low early after pediatric intensive care admission [5]. The role of prolactin as an early acute stress in adult ICU sepsis and trauma patients has not been elucidated yet [6]. In an animal model, a possible relationship between heat shock proteins (HSPs) and prolactin receptors has been previously reported [7].

Intracellular HSPs (iHSPs) are high evolutionary conservative proteins that play an important role in regulating host response against infections, thermal injury, oxidative damage, and hypoxia [8]. Particularly, the major heat shock proteins iHSP70 and iHSP90α confer tolerance to sepsis by maintaining the conformational homeostasis, exerting antiapoptotic effects, and mediating LPS-signaling as a part of the LPS receptor cluster [9]. However, although animal studies have demonstrated a protective effect of iHSP72 in sepsis, human studies are inconclusive showing either protection or relation to mortality and infections [10]. To add more questions about the extracellular HSPs (eHSPs) function and their role in sepsis [11, 12], eHSP90α levels were recently shown to decline in controls and remain increased in septic patients, contrasting eHSP72, which increased over time in both groups [13]. Thus, the involvement of extracellular bound HSPs as signals for activation of the immune system and especially macrophages [14] raises interest about the role of these proteins in sepsis. Apart from septic patients, serum levels of eHSP72 measured early after injury in trauma patients correlated with survival, with significantly higher levels in trauma patients who survived compared to nonsurvivors [15].

In this study, we evaluated the early (first 48 hours) serum levels of prolactin, cortisol, and interleukins and plasma levels of ACTH, eHSP90α, and eHSP72 and measured mean fluorescence intensity (MFI) of iHSP72 and iHSP90α in ICU patients with severe sepsis and septic shock (SS) or systemic inflammatory response syndrome (SIRS) compared to healthy control subjects (*Η*). We also correlated their expression with interleukins (ILs), severity scoring systems, clinical and laboratory data, and outcome.

## 2. Materials and Methods

### 2.1. Patients

The study was approved by the institutional review board of Evangelismos Hospital and was performed during a 14-month period between October 2012 and December 2013. Informed consent form was obtained from the relatives of patients admitted to the ICU. Consecutively admitted patients >18 years and <75 years with early (<48 h) severe sepsis, septic shock, or SIRS admitted to the ICU were eligible for enrolment and were divided in two groups. The SIRS group included trauma patients (*n* = 29) who met at least two of the four conventional criteria for SIRS. The severe sepsis or septic shock group (SS) included patients (*n* = 29) with an identified source of infection. Sepsis, severe sepsis, septic shock, and SIRS were defined according to the Surviving Sepsis Campaign Guidelines [16]. The third group included healthy volunteers (H) (*n* = 16) matched for age and sex to the ICU patients. Exclusion criteria were (a) malignancy, (b) autoimmune diseases, (c) prior use of corticoids, (d) immunosuppressive illness, and (e) late sepsis or SIRS 48 h after admission. Acute physiology and chronic evaluation (APACHE II) [17], sequential organ failure assessment (SOFA) [18], and simplified acute physiology score III (SAPS III) [19] scores were recorded on admission. Demographics, date of hospital, and ICU admission, ICU and in-hospital mortality, length of stay, and laboratory tests were also recorded for all patients.

### 2.2. Laboratory Assays

#### 2.2.1. Prolactin, Cortisol, and ACTH

Blood was drawn between 8 and 9 a.m in the first 48 h after ICU admission. For ACTH measurement, blood was collected into ethylenediaminetetraacetic acid (EDTA) containing tube, immediately centrifuged at 4°C, plasma-pooled, and finally stored at −80°C until measurement. For cortisol and prolactin measurement blood was collected in tubes containing clot and gel for serum separation and centrifuged at 4°C and serum was also stored at −80°C until measurement. Serum cortisol and prolactin levels were determined using the ADVIA Centaur Immunoassay Analyzer (Siemens Healthcare Diagnostics, Tarrytown, NY, USA) while plasma ACTH was measured using the Immulite 2000 Immunoassay Analyzer (Siemens Healthcare Diagnostics, Tarrytown, NY, USA).

#### 2.2.2. Cytokines and Extracellular Heat Shock Proteins

Cytokine levels of serum IL-6, IL-10, IL-17, and IFN-*γ* were measured by ELISA as mentioned by the kit instructions and extracellular plasma levels of HSPs (eHSP72 and eHSP90α) were analyzed by ELISA assay according to the manufacturers' instructions (Invitrogen Carlsbad, CA, USA, and Enzo Life Sciences, Ann Arbor, MI, USA, resp.). The inter- and intra-assay CV for each analyte were as follows: 6.2 and 7.8 for IL-6, 3.25 and 2.75 for IL-10, 3.7 and in process for IL-17, 3.5 and 7.3 for IFN-*γ*, 7.1 and 15.2 for hsp72, and <10 for hsp90α. The sensitivities of the assays were <2 pg/mL for IL-6, <1 pg/mL for IL-10, 2 pg/mL for IL-17, 0.03 IU/mL for IFN-*γ*, 90 pg/mL for hsp72, and 50 pg/mL for hsp90α.

#### 2.2.3. Intracellular HSPs

EDTA-anticoagulated blood (100 *μ*L) was used for flow cytometric analysis of fresh peripheral blood mononuclear cells (PBMCs). Monocytes iHSP72 and iHSP90α expressed as mean fluorescence intensity (MFI) were determined after staining with 5 *μ*L surface antigens CD33-PE/Cy5 (BioLegend, San Diego, CA, USA) and 5 *μ*L CD45 PE/Cy7 (BioLegend, San Diego, USA) followed by either 5 *μ*L HSP72-FITC (Enzo Life Sciences, Ann Arbor, MI, USA) or 5 *μ*L HSP90α-PE (Enzo Life Sciences, Ann Arbor, MI, USA) intracellular staining. Assays were performed according to the manufacturer's instructions using 4-colour flow cytometry FC-500 (Beckman Coulter, Miami, FL, USA).

#### 2.2.4. Statistical Analysis

All results are presented as means ± standard deviation. The results were analyzed using SPSS software (version 21.0, SPSS, Chicago, Ill). Group comparisons were performed using the Kruskal-Wallis test. The variables that showed differences among groups were compared group by group by the Mann-Whitney test. Paired differences for continuous variables in the same subjects were analyzed using the Wilcoxon signed-rank test. The correlation between variables was analyzed by the Spearman correlation test. The level of significance between groups was set on *P* < 0.05.

## 3. Results

Anthropometric characteristics and severity scores of the (H), (SIRS), and (SS) groups are summarized in Table 1. Hormonal profile, extracellular heat shock protein and cytokine measurements, and mean fluorescence intensity of heat shock proteins in monocytes are summarized in Table 2.

**Figure 1:**
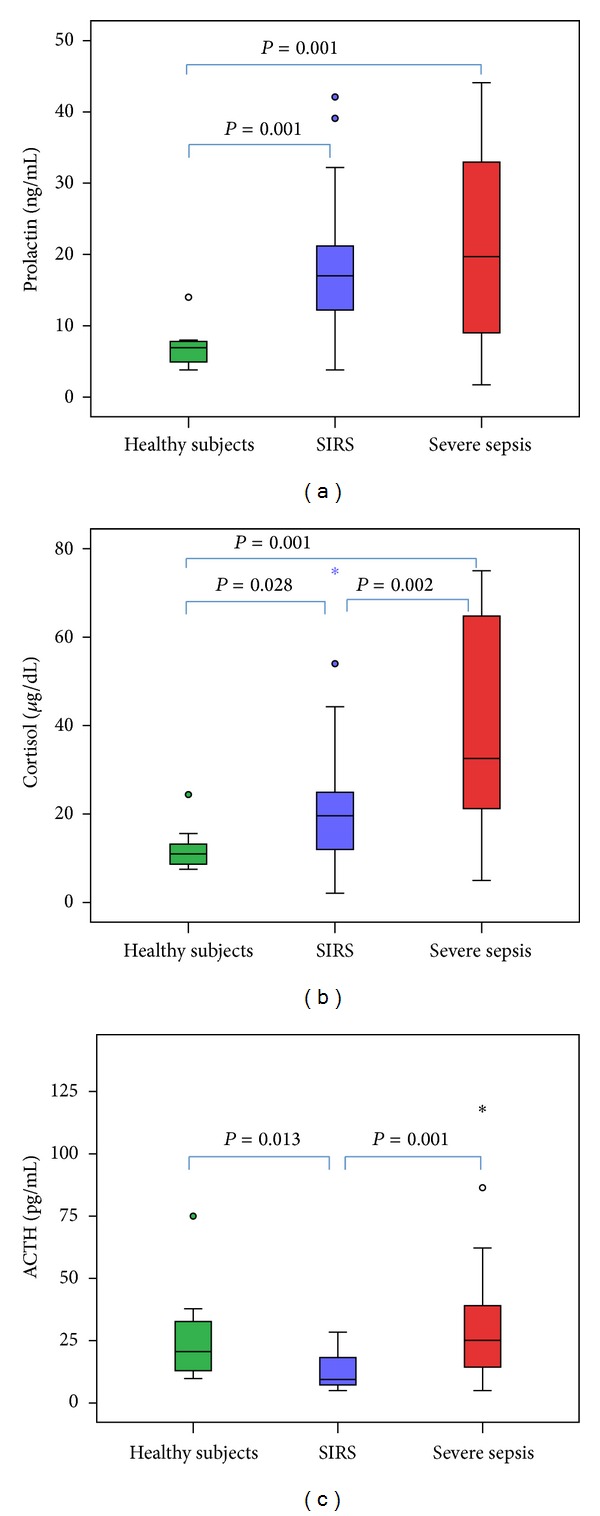
Blood concentrations of (a) prolactin; (b) cortisol; and (c) ACTH for the first 48 hours of ICU admission. The *P* value was calculated using the Mann-Whitney *U* test and a *P* value <0.05 (two sided) was considered statistically significant. The box-whisker plots show the median (horizontal line within the box) and the 10th and 90th percentiles (whiskers). The box length is the interquartile range. Solid circles represent outliers and stars extremes.

Prolactin, cortisol, and ACTH levels differed significantly between groups (Figure 1). Prolactin was correlated with SAPS III (*r* = 0.42, *P* = 0.004) and APACHE II (*r* = 0.3, *P* = 0.04) scores; cortisol was correlated with eHSP90α (*r* = 0.47, *P* = 0.013), IL-6 (*r* = 0.25, *P* = 0.05), and maximum admission day lactate (*r* = 0.30, *P* = 0.03) and negatively with HCO_3_ (*r* = −0.50, *P* = 0.001).

**Figure 2:**
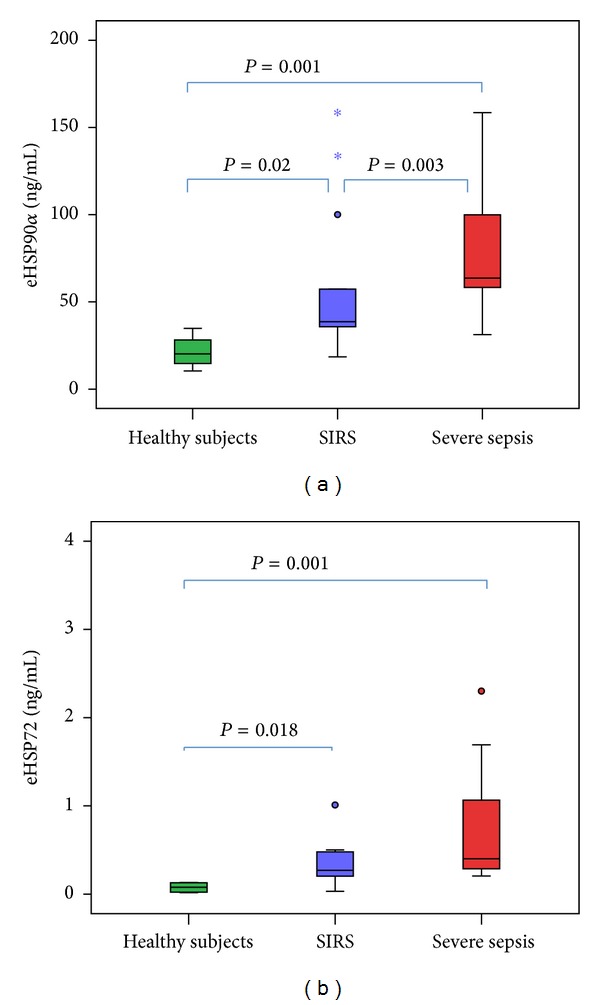
Extracellular heat shock protein (eHSP) levels of (a) eHSP90α and (b) eHSP72 in healthy subjects, SIRS, and severe sepsis patients. The *P* value was calculated using the Mann-Whitney *U* test and a *P* value <0.05 (two sided) was considered statistically significant. The box-whisker plots show the median (horizontal line within the box) and the 10th and 90th percentiles (whiskers). The box length is the interquartile range. Solid circles represent outliers and stars extremes.

The eHSP90α levels in the SS group were increased in comparison to the H and SIRS groups. The eHSP72 levels in the SS group were increased compared to H (Figure 2). Both eHSP90a and eHSP72 were significantly increased in SIRS compared to H (*P* < 0.02).

**Figure 3:**
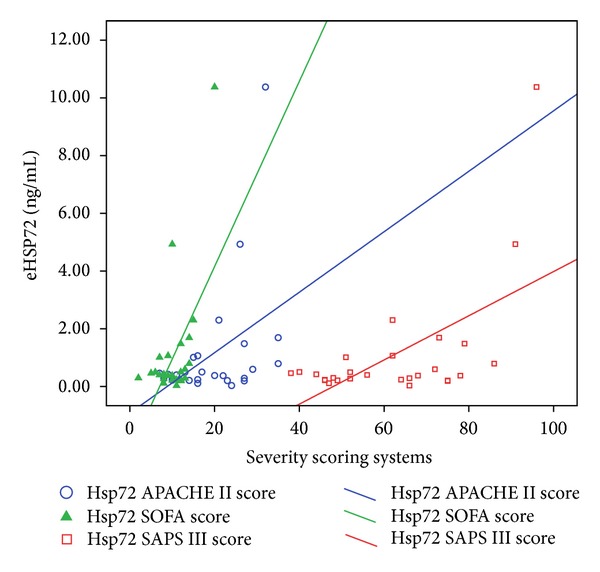
Extracellular heat shock protein (eHSP) levels of eHSP72 in SIRS and sepsis correlated with severity of illness scores APACHE II (*r* = 0.45, *P* = 0.034), SOFA (*r* = 0.6, *P* = 0.002), and SAPS III (*r* = 0.5, *P* = 0.004).

Extracellular HSP72 in the SS and SIRS groups correlated with the severity of illness scores (Figure 3): APACHE II (*r* = 0.45, *P* = 0.034), SOFA (*r* = 0.6, *P* = 0.002), and SAPS III (*r* = 0.5, *P* = 0.004). There was a positive correlation of eHSP72 and eHSP90α levels in all groups (Table 3). The eHSP90α levels showed a positive correlation with IL-6 and IL-10 but not with IL-17 or IF-*γ*. Also, eHSP72 levels were only correlated with IL-10 (Table 3).

Septic monocytes expressed significantly lower iHSP72 MFI levels compared to the H group (Table 2). Although a positive correlation of iHSP90α with iHSP72 monocyte levels was found in the SS and SIRS groups, only iHSP72 correlated negatively with severity of illness scores: Apache II (*r* = −0.32, *P* = 0.004), SOFA (*r* = −0.32, *P* = 0.017), and SAPS III (*r* = −0.34, *P* = 0.012).

## 4. Discussion

In this study we evaluated the early inflammatory and hormonal stress response in SS and SIRS patients in an ICU setting. We showed that in these critically ill patients prolactin levels, along with cortisol, were significantly higher in SS compared to H and SIRS groups. Hormonal increase was related to the severity of illness but only cortisol, not prolactin, was correlated with eHSP90α. Thus, our study demonstrated that stress response of prolactin and its relation to the severity of sepsis might not have been induced through the iHSP72 or iHSP90α “danger signal” pathways.

Patients suffering early sepsis induce elevation of baseline cortisol levels and decrease in blood cortisol to ACTH ratio compared to nonseptic patients admitted to the ICU [20]. Plasma ACTH and prolactin are increased within the few minutes following the insult of a pathogen [2]. Our findings of significantly increased ACTH, cortisol, and prolactin levels in severe sepsis and septic shock also support the hypothesis that prolactin, apart from cortisol, may have a role in the acute stress hormonal response in the early-onset inflammatory process. It has been previously suggested that, with nitric oxide as a key mediator, sepsis elicits a very reproducible pattern of pituitary hormone secretion, with plasma ACTH and prolactin increasing within a few minutes following the insult and with a rapid inhibition of secretion of luteinizing and thyroid-stimulatory hormone [2]. This is further supported by a strongly positive relationship of prolactin with the evaluated in this study's severity scoring systems.

Cortisol and ACTH levels have extensively been studied in a critical ill setting. On the contrary, prolactin's role as an immunomodulator in septic patients needs to be further elucidated. A recent study has suggested an association of increased prolactin mRNA expression in monocytes with better outcome in hemato-oncological septic patients [21]. It has been also found that anterior pituitary cells recognize and respond to fungal cell wall glucans by appropriately stimulating the secretion of prolactin, a hormone that plays an important role in the response to fungal infection [22]. In addition, the increased prolactin levels in our trauma patients might further suggest that the prolactin's response role is equally important to that of cortisol in homeostasis not only in infectious but also in noninfectious SIRS.

Although none of the eHSPs correlated with prolactin levels, cortisol was correlated with eHSP90α. It has been previously shown that eHSP90α plays a crucial role in antigen presenting in dendritic cells through the major histocompatibility complex (MHC) type 1 [23] and stimulates monocytes [24]. To the best of our knowledge, this is the first time that a significant early increase of plasma eHSP90α is shown in septic patients compared to healthy subjects and severely injured patients. In addition, the strong correlation of eHSP90α with eHSP72 found in our study might further suggest a common pathophysiologic mechanism of their expression. Thus, extracellular levels of both HSPs were significantly elevated in severely septic patients as compared to healthy volunteers; moreover, eHSP90α levels of SS were higher when compared to trauma (SIRS) patients. Importantly, in both SS and SIRS groups, eHSP72 levels correlated with the severity of illness, further supporting the hypothesis that eHSPs may act as danger signals to modulate the immune system [8].

Increased eHSP72 levels have been found in inflammatory myopathy [25], lung injury [26], acute coronary syndrome and stable angina [27], trauma [15], inflammation, and sepsis [12]. Only two in vivo studies of eHSP72 and none of eHSP90α were identified in a PubMed database research in human severe sepsis (1992–2012) [12, 28]. Wheeler et al. [28] found that serum levels of eHSP72 in children with septic shock were significantly elevated as compared to noncritically ill children undergoing elective surgical procedures and that the increase was related to mortality. Analysis of oxidative parameters in septic patients by Gelain et al. revealed that serum levels of eHSP72 were modulated according to oxidative stress [12]. In our study we further showed a significant early increase of eHSP72 in both septic and traumatic adult patients compared to controls.

It has been suggested that stimulation of toll-like receptors (TLR)-dependent signaling pathways by lipopolysaccharides (LPS) increases iHSP72 expression and its release in the extracellular environment both in in vitro cell cultures and in vivo [29]. The release of eHSPs appears to be a very complex phenomenon encompassing different alternative pathways containing nonconsensus secretory signals [30]. Proposed mechanisms for their release are translocation across the plasma membrane, release associated with lipid vesicles, passive release after cell death by necrosis via extracellular vesicles [11], and through nonclassical exomal nonsecretory pathways [31]. Although in septic patients stimulation of LPS pathways has been proposed to explain the extracellular release of eHSPs, it might not clarify the increased eHSPs levels in our SIRS patients. Instead, it has been hypothesized that eHSPs may be released from damaged tissue and necrotic cells after trauma contributing to trauma induced immunosuppression [32].

In severe sepsis, in contrast to the extracellular monocyte-HSPs, intracellular monocyte-HSPs had a tendency to decrease in comparison to control subjects. These findings are in accordance with those of another prospective observational study in patients with severe sepsis demonstrating a reduced iHSP72 expression in PBMCs and a LPS dose-dependent inhibition of its expression [33]. In patients with inflammation, however, iHSPs were found increased compared to healthy control subjects [34]. Although SIRS was shown to increase iHSP72 and iHSP90α expression in monocytes indicating a protective function of iHSPs during the acute phase of stress, the downregulation of iHSP-positive cells in SS seems to be a result of (mal-)adaptation mechanism to severity of illness [35]. Preliminary data showed that iHSP72 and iHSP90α monocyte expressions follow different longitudinal courses in SS, not related to the metabolic response to stress [36].

The main limitations of our study are the small number of patients and an age difference among groups. Enrollment of septic patients has been proven to be difficult since most of them met exclusion criteria (immunosuppression, cancer). In addition, since that was a prospective study with consecutively admitted patients in ICU, the mean age of trauma patients was, as expected, lower than the mean age of septic patients. The mean age of healthy subjects, however, was matched with the mean age of ICU patients included in our study. Finally, we did not find any correlation between hormones and age, so that the importance of the age difference might not have significantly influenced results.

## 5. Conclusions

Prolactin, apart from cortisol, may have an early role in the acute stress hormonal response in severe sepsis and septic shock. Extracellular levels of HSP72 and HSP90α are significantly elevated in septic patients as compared to healthy volunteers supporting the hypothesis that eHSPs may act as danger signals to activate the immune system. Simultaneously, however, monocytes suppress iHSP72 expression early in septic patients. To the best of our knowledge, this is the first time that a significant early increase of plasma eHSP90α is shown in septic patients compared to healthy subjects and severely injured patients and that this increase is related to increased cortisol levels.

## Figures and Tables

**Table 1:** Demographic characteristics and severity scores by group (healthy subjects *n* = 16, SIRS *n* = 29, severe sepsis *n* = 29)∗∗.

	Healthy subjects	SIRS	Severe sepsis	*P* value∗
Age (years)	47.3 ± 9.7	42.5 ± 13.0	54.7 ± 14.5^ab^	0.003
Gender (male/female)	10 M/6 F	24 M/5 F	17 M/12 F	0.118
APACHE II		15.0 ± 5.4	22.0 ± 9.7^b^	0.007
SOFA		8.0 ± 3.0	11.0 ± 4.0^b^	0.006
SAPS III		50.0 ± 10.0	68.0 ± 14.0^b^	0.0001

APACHE: acute physiology and chronic evaluation; SOFA: sequential organ failure assessment; SAPS III: simplified acute physiology score; SIRS: systematic inflammatory response syndrome, ^a^ *P* < 0.05 between healthy subjects and severe sepsis groups, and ^b^ *P* < 0.05 between SIRS and severe sepsis group.

∗The *P* value was calculated using the Kruskal-Wallis test and a *P* value <0.05 was considered statistically significant.

∗∗Data are expressed as means ± SD (standard deviation).

**Table 2:** Hormonal, cytokine, and heat shock protein measurements by group (healthy subjects *n* = 16, SIRS *n* = 29, severe sepsis *n* = 29)^∗∗^.

	Healthy subjects	SIRS	Severe sepsis	*P* value∗
Prolactin (ng/mL)	5.97 ± 2.80	18.26 ± 10.64^a^	23.17 ± 18.38^b^	0.003
Cortisol (*μ*g/dL)	12.07 ± 5.03	21.74 ± 16.09^a^	39.64 ± 23.52^bc^	0.000
ACTH (pg/mL)	25.97 ± 19.05	30.71 ± 69.54^a^	31.26 ± 25.18^c^	0.002
eHSP90*α* (ng/mL)	21.50 ± 10.08	58.70 ± 44.3^a^	81.89 ± 41.81^bc^	0.003
eHSP72 (ng/mL)	0.07 ± 0.06	0.35 ± 0.26^a^	1.43 ± 2.76^b^	0.005
iHSP90*α* (MFI)	49.73 ± 30.87	41.20 ± 31.62	37.36 ± 28.96	0.490
iHSP72 (MFI)	38.08 ± 24.76	36.65 ± 31.38	24.66 ± 21.15^b^	0.093
IL-6 (pg/mL)	1.70 ± 1.50	188.10 ± 203.92^a^	358.49 ± 377.40^b^	0.001
IL-10 (ng/mL)	0.01 ± 0.001	5.16 ± 7.10	35.29 ± 60.60^b^	0.037
IL-17 (ng/mL)	9.75 ± 16.65	20.86 ± 60.96	25.14 ± 93.56	0.969
IFN-*γ* (pg/mL)	0.52 ± 1.07	0.19 ± 0.10	0.92 ± 1.89^c^	0.070

eHSP: extracellular heat shock protein; iHSP: intracellular heat shock protein; IL: interleukins; IFN-*γ*: interferon gamma.

^a^ *P* < 0.05 between healthy subjects and SIRS groups.

^b^ *P* < 0.05 between healthy subjects and severe sepsis groups.

^c^ *P* < 0.05 between SIRS and severe sepsis group.

∗The *P* value was calculated using the Kruskal-Wallis test and a *P* value <0.05 was considered statistically significant.

∗∗Data are expressed as means ± SD (standard deviation).

**Table 3:** Pearson's coefficient correlations of hormones, extracellular heat shock proteins, and interleukins.

Prolactin		Prolactin	Cortisol	ACTH	eHSP90α	eHSP72	IL-6	IL-10	IL-17
Cortisol	*r*	**0.24**							
	*p*	**0.05**							

ACTH	*r*	−0.1	0.05						
	*p*	0.3	0.68						

eHSP90α	*r*	0.09	**0.47**	0.02					
	*p*	0.62	**0.013**	0.9					

eHSP72	*r*	0.35	0.26	−0.02	**0.553**				
	*p*	0.08	0.23	0.95	**0.002**				

IL-6	*r*	0.08	0.24	0.02	**0.5**	−0.01			
	*p*	0.5	0.05	0.8	**0.007**	0.9			

IL-10	*r*	0.16	0.2	−0.07	**0.6**	**0.7**	**0.27**		
	*p*	0.3	0.2	0.60	**0.001**	**0.000**	**0.06**		

IL-17	*r*	−0.11	0.02	−0.080	−0.136	−0.007	−0.4	−0.08	
	*p*	0.48	0.90	0.596	0.467	0.97	0.3	0.58	

IFN-*γ*	*r*	−0.07	0.195	−0.05	0.04	−0.067	0.08	**0.45**	−0.05
	*p*	0.58	0.125	0.69	0.84	0.733	0.5	**0.02**	0.7

eHSP: extracellular heat shock protein; iHSP: intracellular heat shock protein; IL: interleukins; IFN-*γ*: interferon gamma.

## References

[1] Angus DC, Linde-Zwirble WT, Lidicker J, Clermont G, Carcillo J, Pinsky MR (2001). Epidemiology of severe sepsis in the United States: Analysis of incidence, outcome, and associated costs of care. *Critical Care Medicine*.

[2] Maxime V, Siami S, Annane D (2007). Metabolism modulators in sepsis: the abnormal pituitary response. *Critical Care Medicine*.

[3] Kanczkowski W, Alexaki V, Tran N (2013). Hypothalamo-pituitary and immune-dependent adrenal regulation during systemic inflammation. *Proceedings of the National Academy of Sciences of the United States of America*.

[4] Oberbeck R, Schmitz D, Wilsenack K (2003). Prolactin modulates survival and cellular immune functions in septic mice. *Journal of Surgical Research*.

[5] Heidemann SM, Holubkov R, Meert KL (2013). Baseline serum concentrations of zinc, selenium, and prolactin in critically ill children. *Pediatric Critical Care Medicine*.

[6] Dossett LA, Swenson BR, Evans HL, Bonatti H, Sawyer RG, May AK (2008). Serum estradiol concentration as a predictor of death in critically ill and injured adults. *Surgical Infections*.

[7] Saribek B, Jin Y, Saigo M, Eto K, Abe S (2006). HSP90*β* is involved in signaling prolactin-induced apoptosis in newt testis. *Biochemical and Biophysical Research Communications*.

[8] McConnell KW, Fox AC, Clark AT (2011). The role of heat shock protein 70 in mediating age-dependent mortality in sepsis. *Journal of Immunology*.

[9] Ambade A, Catalano D, Lim A, Mandrekar P (2012). Inhibition of heat shock protein (molecular weight 90 kDa) attenuates proinflammatory cytokines and prevents lipopolysaccharide-induced liver injury in mice. *Hepatology*.

[10] Briassoulis G, Briassouli E, Fitrolaki DM (2014). Heat shock protein 72 expressing stress in sepsis: unbridgeable gap between animal and human studies—A Hypothetical “Comparative” study. *BioMed Research International*.

[11] De Maio A, Vazquez D (2013). Extracellular heat shock proteins: a new location, a new function. *Shock*.

[12] Gelain DP, De Bittencourt Pasquali MA, M. Comim C (2011). Serum heat shock protein 70 levels, oxidant status, and mortality in sepsis. *Shock*.

[13] Briassouli E, Goukos D, Daikos G (2014). Glutamine suppresses Hsp72 not Hsp90α and is not inducing Th1, Th2, or Th17 cytokine responses in human septic PBMCs. *Nutrition*.

[14] Vega VL, Rodríguez-Silva M, Frey T (2008). Hsp70 translocates into the plasma membrane after stress and is released into the extracellular environment in a membrane-associated form that activates macrophages. *Journal of Immunology*.

[15] Pittet J, Lee H, Morabito D, Howard MB, Welch WJ, Mackersie RC (2002). Serum levels of Hsp 72 measured early after trauma correlate with survival. *The Journal of Trauma*.

[16] Dellinger RP, Levy MM, Rhodes A (2013). Surviving sepsis campaign: international guidelines for management of severe sepsis and septic shock: 2012. Surviving Sepsis Campaign Guidelines Committee including the Pediatric Subgroup. *Intensive Care Medicine*.

[17] Knaus WA, Draper EA, Wagner DP, Zimmerman JE (1985). APACHE II: a severity of disease classification system. *Critical Care Medicine*.

[18] Vincent J-L, Moreno R, Takala J (1996). The SOFA (Sepsis-related Organ Failure Assessment) score to describe organ dysfunction/failure. *Intensive Care Medicine*.

[19] Moreno RP, Metnitz PGH, Metnitz B, Bauer P, Afonso de Carvalho S, Hoechtl A (2008). Modeling in-hospital patient survival during the first 28 days after intensive care unit admission. A prognostic model for clinical trials in general critically ill patients. *The Journal of Critical Care*.

[20] Lesur O, Roussy J, Chagnon F (2010). Proven infection-related sepsis induces a differential stress response early after ICU admission. *Critical Care*.

[21] Cejková P, Chromá V, Cerná M (2012). Monitoring of the course of sepsis in hematooncological patients by extrapituitary prolactin expression in peripheral blood monocytes. *Physiological Research*.

[22] Breuel KF, Kougias P, Rice PJ (2004). Anterior pituitary cells express pattern recognition receptors for fungal glucans: implications for neuroendocrine immune involvement in response to fungal infections. *NeuroImmunoModulation*.

[23] Oura J, Tamura Y, Kamiguchi K (2011). Extracellular heat shock protein 90 plays a role in translocating chaperoned antigen from endosome to proteasome for generating antigenic peptide to be cross-presented by dendritic cells. *International Immunology*.

[24] Cecchini P, Tavano R, Polverino de Laureto P (2011). The soluble recombinant Neisseria meningitidis adhesin NadA *δ*351-405 stimulates human monocytes by binding to extracellular Hsp90. *PLoS ONE*.

[25] Svitalkova T, Remakova M, Plestilova L (2014). Plasma level of HSP70 protein is increased in czech patients with idiopathic inflammatory myopathy. *Annals of the Rheumatic Diseases*.

[26] Ganter MT, Ware LB, Howard M (2006). Extracellular heat shock protein 72 is a marker of the stress protein response in acute lung injury. *The American Journal of Physiology—Lung Cellular and Molecular Physiology*.

[27] Zhang X, Xu Z, Zhou L (2010). Plasma levels of Hsp70 and anti-Hsp70 antibody predict risk of acute coronary syndrome. *Cell Stress and Chaperones*.

[28] Wheeler DS, Fisher LE, Catravas JD, Jacobs BR, Carcillo JA, Wong HR (2005). Extracellular hsp70 levels in children with septic shock. *Pediatric Critical Care Medicine*.

[29] Gupta A, Cooper ZA, Tulapurkar ME (2013). Toll-like receptor agonists and febrile range hyperthermia synergize to induce heat shock protein 70 expression and extracellular release. *Journal of Biological Chemistry*.

[30] de Maio A (2011). Extracellular heat shock proteins, cellular export vesicles, and the Stress Observation System: a form of communication during injury, infection, and cell damage: It is never known how far a controversial finding will go! Dedicated to Ferruccio Ritossa. *Cell Stress and Chaperones*.

[31] Li W, Sahu D, Tsen F (2012). Secreted heat shock protein-90 (Hsp90) in wound healing and cancer. *Biochimica et Biophysica Acta—Molecular Cell Research*.

[32] Flohé SB, Bangen JM, Flohé S, Agrawal H, Bergmann K, Schade FU (2007). Origin of immunomodulation after soft tissue trauma: Potential involvement of extracellular heat-shock proteins. *Shock*.

[33] Schroeder S, Bischoff J, Lehmann LE (1999). Endotoxin inhibits heat shock protein 70 (HSP7O) expression in peripheral blood mononuclear cells of patients with severe sepsis. *Intensive Care Medicine*.

[34] Njemini R, Bautmans I, Lambert M, Demanet C, Mets T (2007). Heat shock proteins and chemokine/cytokine secretion profile in ageing and inflammation. *Mechanisms of Ageing and Development*.

[35] Apostolou KT, Psara K, Briassouli E (2013). Heat shock protein 72 and 90 intracellular monocyte expression in patients with sepsis or sirs: preliminary data. *Intensive Care Medicine*.

[36] Tavladaki T, Spanaki AM, Dimitriou H (2013). Expression of intracellular HSPs in neutrophil and monocyte subjects during acute phase of stress in critical ill patients: preliminary data. *Intensive Care Medicine*.

